# Medication information completeness in discharge summaries from a Norwegian rural hospital – a cross-sectional study

**DOI:** 10.1186/s12913-025-12669-x

**Published:** 2025-05-01

**Authors:** Beate Hennie Garcia, Michelle Thao Nguyen, Lars Småbrekke, Frode Skjold, Trine Aag

**Affiliations:** 1 Faculty of Health Sciences, UiT the Arctic University of Norway, Tromsoe, 9037 Norway; 2 Hospital Pharmacy of North Norway Trust, Postboks 6147, Langnes, Tromsø, 9291 Norway; 3 Helgelandssykehuset Mo i Rana, Postboks 601, Mo i Rana, 8607 Norway

**Keywords:** Medication information, Discharge summary, Audit, Completeness, Quantile regression

## Abstract

**Background:**

Hospital discharge summaries are crucial for transferring patient information to subsequent care providers, yet they often contain incomplete and incorrect medication details. This may lead to inappropriate medication therapy, medication-related problems and unnecessary patient harm. A 2014 study in Norway highlighted a low level of medication information completeness at a rural hospital. This study aimed to audit the completeness of medication information in discharge summaries from the same hospital and to identify factors that could improve medication safety in future efforts.

**Methods:**

We randomly selected 240 discharge summaries from 2019 and applied seven national criteria defining the necessary medication information in discharge summaries; (1) reasons for changes in medication prescribing during hospitalization, (2) generic names, (3) administration forms, (4) dosage strengths, (5) dosage regimes stated, (6) indications for use and (7) the medication status categories new, changed, short course. A quantile regression model was applied to analyze factors associated with the medication information completeness in these summaries, adjusting for both patient- and hospital-related variables.

**Results:**

From 2550 assessed medications, information completeness in discharge summaries ranged from 0.0 to 1.0, with a mean of 0.904 (SD 0.15). The criteria with lowest information completeness were ´indication for use´ and ´reasons for changes in medication use stated at discharge´. A significant factor in enhancing completeness was the use of a digital tool for compiling the medication list, which increased the completeness coefficient by 0.23 to 0.83 when applied.

**Conclusions:**

The completeness of medication information in discharge summaries from Helgelandssykehuset Mo i Rana was high and has significantly improved since 2014. The use of electronic tools for compiling medication lists notably enhances information completeness, while free-text lists should be avoided. This should be considered when developing future electronic medications management systems and tools to ensure quality of medication information.

**Supplementary Information:**

The online version contains supplementary material available at 10.1186/s12913-025-12669-x.

## Background

Inadequate communicate medication information in hospital discharge summaries may increase the risk of post discharge prescribing errors, failure to implement appropriate drug monitoring, and unplanned rehospitalisation [1–3]. Key factors contributing to poor quality of discharge summaries include delayed submission to the next care level, discharge letters that are not patient centered, lack of information, and lack of training in writing discharge letters [4].

A systematic review on the prevalence and nature of medication errors and medication-related harm revealed that nearly 50% of adult and elderly patients experienced medication errors or unintentional medication discrepancies after hospital, with nearly 20% affected by adverse drug events post- discharge [5]. Ensuring accurate and complete information in discharge summaries is crucial for preventing misunderstandings and incorrect prescribing at the next care level, reducing drug-related problems, and decreasing the likelihood of unplanned rehospitalizations, morbidity, and mortality [1–3].

In 2013, Hammad et al. audited discharge summaries across six hospitals in the United Kingdom, assessing information completeness regarding dose, frequency, route of administration, formulations, therapy duration for medications initiated at the hospital, as well as explanations for any therapy alterations during hospitalization [6]. They fond 67% adherence to criteria concerning dosage, strength and dosage intervals, but only 49% adherence to criteria concerning therapy changes [6]. In 2020, Shah et al. applied similar criteria as the national minimum standards for medication-related information on discharge summaries in United Kingdom (including allergies, changes to medication regimen, minimum prescription standards such as dose, route, formulation and duration, along with medication reconciliation (MedRec)) to evaluate 10 038 prescribed medications in 1454 discharge summaries [7]. They found that while most medication details complied with standards, compliance was low for indication (12%), formulation (60%) and instructions of ongoing use (73%). Additionally, documentation completeness was only 49%, 39% and 57% for newly started medications, dose changes and discontinued medications, respectively. A 2017 audit in Australia identified only 46% accuracy in compliance with national standards, with most errors involving omissions of required medications. Notably, none of the discharge summaries included documented reasons for medication changes [8].

In Norway, explicit criteria for evaluating medication information completeness in discharge summaries were established in 2011, with an updated version released in 2015 [9]. These criteria are similar to criteria applied by Hammad and Shah et al. [6, 7]. The only two Norwegian studies investigating medication information completeness in discharge summaries are by Frydenberg and Brekke in 2011 [10], and Garcia et al. in 2017 [11]. While Frydenberg and Brekke reported high information completeness, Garcia et al. found very low information completeness, particularly lacking details about generic names, indications for medication use, explanations for medication changes, and the source of information. Both studies, however, noted a low documentation rate regarding medication changes. Only Garcia et al. applied the national criteria, which includes the criterion requesting documentation of medication changes [9]. The low medication completeness identified by Garcia et al. may have been influenced by the absence of an electronic medication management system (EMMS) at the hospital. Even today, numerous Norwegian hospitals lacka fully operational EMMS.

Encouragingly, the focus on medication completeness and accuracy has been increased in Norway, supported by MedRec procedures, training programs, EMMS and digital tools to compile medication lists [12]. Additionally, “The Patient’s Medication List”, aimed at electronically share updated medication information throughout the entire patient trajectory across organizations, is one of the prioritized areas in the Norwegian e-health strategy [13]. Similar processes have been ongoing world-wide [14, 15].

## Methods

### Aim

The aim of this study was to audit the medication information completeness in discharge summaries from the same rural hospital as in 2014. Additionally, to guide future efforts aimed at enhancing medication safety, we explored factors associated with medication information completeness.

### Study design and setting

This is a retrospective cross-sectional study using data from patient records from a Norwegian rural hospital; Helgelandssykehuset Mo i Rana. The hospital is located in central Norway and had about 42 000 somatic hospital admissions in 2019. According to local procedures, medication lists are electronically updated upon hospital admission, but handwritten paper charts are compiled for use during hospitalization. Upon discharge, electronic medication lists are updated before electronically transferred into discharge summaries. Discharge summaries are compiled by physicians and sent electronically to the next care provider after discharge. Discharge summaries are stored in the electronic patient journal and the patient receives a printed copy at discharge or alternatively by mail.

### Inclusion and exclusion criteria

We included discharge summaries from 2019 along with corresponding admission notes and hand-written medication charts, randomly selecting 20 discharge summaries for each month, totaling 240 (7.3% of all discharge summaries from 2019). The sample size was deemed manageable, for data collection by a single person within the allotted study period. We included discharge summaries for patients who had been fully admitted from the Department of Medicine and Surgery, representing most of the hospital´s patient population. We excluded discharge summaries from the Psychiatric Department and the Maternity ward, which would enable comparison with the 2014 study [16]. We also excluded discharge summaries for patients not using medications, those with missing medication list in the admission report or missing admission report, patients who died during hospitalization, and patients transferred to other hospitals/departments. For randomization, discharge notes were sequentially numbered each month and by department, and the Research Randomizer was used to select the notes for the audit [17]. As patient data were unavailable during randomization, blinding was unnecessary.

### Data collection and assessment of medication completeness

Data collection was performed during September – December 2019. For each discharge summary, we collected data on MedRec and source of medication information, patient demographics, details about the hospitalization, and the medications used by the patient at admission and at discharge, along with any comments made and recommendations stated.

All medications prescribed for use after discharge were individually assessed based on seven criteria defined by the Norwegian Safety Program, which outlines the necessary medication information that should be included in Norwegian hospital discharge summaries [12]. These criteria are: 1) reasons for changes in medication prescribing during hospitalization, 2) generic names, 3) administration forms, 4) dosage strengths, 5) dosage regimes stated, 6) indications for use and 7) the medication status categories *new*,* changed*,* short course* (stated behind each new or changed medication). These criteria are designed to ensure that all necessary information is provided to facilitate safe medication use post-discharge and to enable continuity of care.

All criteria were assessed yes (= 1) or no (= 0), depending on whether the information was present or not. Medications discontinued during hospitalization were also assessed with regards to criterion 1, which involves documenting reasons for changes in medication prescribing during hospitalization. To ensure consistency, we developed an application guide after reviewing the first 10 discharge summaries. This guide, refined through team discussions and integrated conclusions, was actively used during data collection to standardize the assessment of each discharge summary against the defined criteria. This approach helped to minimize variability and enhance the reliability of the data collected. Blinding of the assessor was not possible.

### Outcome measures

We calculated a “proportion of information completeness”, both on medication level and discharge summary level. E.g., if all information requested for one medication, the proportion of available information was 1.0. If only half of the information was available, the proportion of available information was 0.5. We excluded non-applicable criteria from the equation, e.g., criterion 1 was not applicable if no changes in medications had been made. The main outcome measures was the proportion of information available on a discharge summary level, calculated by summarizing all scores for information available and presenting this as a proportion of information assumed to be available.

### Data presentation and statistics

For data management and analyses we applied Microsoft^®^ Excel for Mac version 16.69.1, IBM^®^ SPSS Statistics version 29.0.0.0 (241) and R (http://www.R-project.org). Results are expressed with means, proportions, standard deviations (SDs), minimum and maximum values and 95% confidence intervals where appropriate. The significance level in all analyses were set to *p* < 0.05.

To investigate intra- and inter-rater agreement, we randomly selected 24 (10%) discharge summaries which was scored a second time after 9 months by the main rater (MT) and by one of the authors (TA), respectively. We calculated inter- and intra-rater agreement by applying Cohen´s kappa (κ) statistics. According to Robson, agreement is considered as following: κ ≥ 0.75 excellent, κ = 0.6–0.75 good, κ = 0.4–0.6 satisfactory, while κ < 0.4 poor [18].

To explore factors influencing the outcome “information completeness” on a discharge summary level”, we used a quantile regression. Quantile regression enables modelling of the relationship between the predictor variables across different quantiles of the response variable [19]. This approach allowed an examination of variables at various levels of information completeness, ranging from the 10th percentile (discharge summaries with low medication completeness) to the 75th percentile (discharge summaries with high medication completeness). We applied a model with four quantiles, 10th percentile, 25th percentile, 50th percentile, 75th percentile, as adviced by Staffa et al. in exploratory studies [20]. The 90th percentile could not be included as the outcome in this quantile was close to 100%. Variables included in the regression model were: sex and age of patient, number of medications in discharge summary, whether the patient had been admitted to hospital in the previous 30 days, whether the medication list was reconciled at admission, whether the patient was admitted to a medical or surgical ward, whether the patient was admitted from home or institution, whether the admission was planned or acute, and whether an electronic system for compiling the medication list was applied at discharge. Thirty seven patients (15%) had missing data for living status before admission. After multiple imputation based on the dataset variables, status changed to ´home´ for 35 patients and to ´not home´for two patients. Regression on imputed data resulted in minor changes in the estimates and the precision of the coefficients [21].

### Ethics approval and consent to participate

The study was conducted in compliance with the Declaration of Helsinki. All experimental protocols were approved by the Ethics Committee at the Hospital of Helgeland (Helgelandssykehuset), project number 08–19. To protect patient confidentiality, all sensitive patient data was managed within the hospital system. Only final scores and anonymized patient characteristics were exported for analysis purposes. The study is reported according to STROBE statement checklist for *cross-sectional studies* [22].

## Results

### Patients, hospitalizations and discharge summaries

Of the 240 included discharge summaries, 166,69.2% of the patients were admitted to medical departments. The age of the patients ranged between 4 and 101 years, and 130, 54.2% were male. A total of 225, 93.8% of the medication lists were compiled with electronic medication module at discharge, and 91, 37.9% were indicated as reconciled at discharge. See Table 1.

**Table 1:** Characteristics of patients, discharges summaries and hospitalizations (*n* = 240)

	0–10 percentile (*n* = 24)		10–25 percentile (*n* = 37)		25–50 percentile (*n* = 59)		50–75 percentile (*n* = 60)		75–100 percentile (*n* = 60)		Total (*n* = 240)	
**Age, years**												
Mean, SD	57.5	22.2	70.2	16.6	71.7	20.2	73.0	15.3	67.3	17.4	69.3	18.4
Min/max values	6	90	34	97	10	95	27	101	4	88	4	101
**Sex**												
Male sex, n, %	14	58.3	20	54.1	28	47.5	37	61.7	31	51.7	130	54.2
**Living situation before admission**												
Home, n, %	12	50.0	21	56.8	38	64.4	38	63.3	43	71.7	152	63.3
Not home^a^, n, %	2	8.3	9	24.3	16	27.1	16	26.7	8	13.3	51	21.3
Unknown, n, %	10	41.7	7	18.9	5	8.5	6	10.0	9	15.0	37	15.4
**Type of admission**												
Emergency, n, %	21	87.5	36	97.3	49	83.1	52	86.7	49	81.7	208	86.6
Planned^b^, n, %	2	8.3	1	2.7	10	16.9	8	13.3	11	18.3	32	13.4
**Type of department admitted to**												
Surgical, n, %	12	50.0	10	27.0	16	27.1	16	26.7	20	33.3	74	30.8
Medical, n, %	12	50.0	27	73.0	43	72.9	44	73.3	40	66.7	166	69.2
**Length of stay in hospital**,** days**												
Mean, SD	3.2	3.9	6.1	6.5	5.1	4.4	4.6	4.1	3.5	5.3	4.5	5.0
Min/max values	0.0	17.0	0.0	25.0	0.0	19.0	0.0	18.0	0.0	37.0	0	37
**Previous hospital admissions**												
≤ 30 days, n, %	0	0	3	8.1	10	16.9	6	10.0	8	13.3	27	11.3
≤ 90 days, n, %^c^	3	12.5	9	24.3	21	35.6	20	33.3	10	16.7	63	26.3
**MedRec performed**												
Reconciled at hospital admission, n, %	20	83.3	32	86.5	57	96.6	56	93.3	57	95.0	222	92.5
Reconciled at hospital discharge, n, %	8	33.3	11	29.7	21	35.6	23	38.3	28	46.7	91	37.9
**Compilation of med list at admission**												
Electronic tool, n, %	14	58.3	29	78.4	55	93.2	58	96.7	55	91.7	211	87.9
Free text, n, %	4	16.7	1	2.7	1	1.7	0	0	3	5.0	9	3.8
Do not use medications^d^, n, %	6	25.0	5	13.5	1	1.7	1	1.7	1	1.7	14	5.8
No medication list^e^, n, %	0	0	2	5.4	2	3.4	1	1.7	1	1.7	6	2.5
**Compilation of med list at discharge**												
Electronic tool, n, %	12	50.0	35	94.6	58	98.3	60	100.0	60	100.0	225	93.8
Free text, n, %	9	37.5	2	5.4	1	1.7	0	0	0	0	12	5.0
No medication list^f^, n, %	3	12.5	0	0	0	0	0	0	0	0	3	1.3
**No of meds in discharge summaries**												
**Total**, n, %	121	5.0	321	13.3	673	27.9	710	29.4	587	24.3	2412	100
Mean, SD	5.3	4.6	8.7	5.2	11.4	6.0	11.8	5.0	9.8	6.1	10.1	5.9
Min/max	1	20	1	21	2.00	22	3	24	1	28	1	28
**Used regularly**, n, %	70	57.8	176	54.8	403	59.9	441	62.1	356	60.7	1446	60.0
Mean, SD	2.9	3.5	4.8	3.8	6.8	3.9	7.4	3.6	5.9	3.9	6.0	4.0
Min/max	0	13	0.00	16	0	15	0	19	0	15	0	19
**Used as needed**, n, %	38	31.4	102	31.8	200	29.7	188	26.5	173	29.5	701	29.0
Mean, SD	1.6	1.7	2.8	2.3	3.4	2.7	3.1	2.3	2.9	2.6	2.9	2.5
Min/max	0	7	0.00	9	0	11	0	11	0	10	0	11
**Used for a short period of time**, n, %	13	10.7	43	13.4	70	10.4	81	11.4	58	9.9	265	11.0
Mean, SD	0.5	1.1	1.2	1.4	1.2	1.4	1.4	1.5	1.0	1.3	1.1	1.4
Min/max	0	4	0.00	5	0	5	0	6	0	7	0	7

*Med* medication, *Med Rec* Mediation Reconciliation, *SD* standard deviation

^a^ institution, nursing home or home care facility

^b^ indicated in the hospital system, e.g., a planned hip surgery

^c^ number also included the ≤ 30 days

^d^ the discharge summary comprised information about medications

^e^ the discharge summary comprised a medication list

^f^ the admission note comprised a medication list

The discharge summaries contained 2412 medications. The mean number of medications used at discharge was 10.1 (SD 6.0), with a range of 1 to 28. Of the medications used at discharge, 1446, 60% were used regularly, 701, 29% were used as needed and 265, 11% were to be used for a short time.

### Medication information completeness

We evaluated 2520 medications, which exceeds the number stated in the discharge summaries, as 108, 4.3% medications were discontinued during hospitalization. We applied 15,483 criteria, as 2157, 12.2% were deemed non-applicable. The non-applicable criteria primarily concerned critera 3–6 for 108 medications stopped during hospitalization, as this information is not requested for discontinued medication. Additionally, criterion 1 was non-applicable for 1717, 68.2% medications that had remained unchanged during hospitalization.

**Fig. 1:**
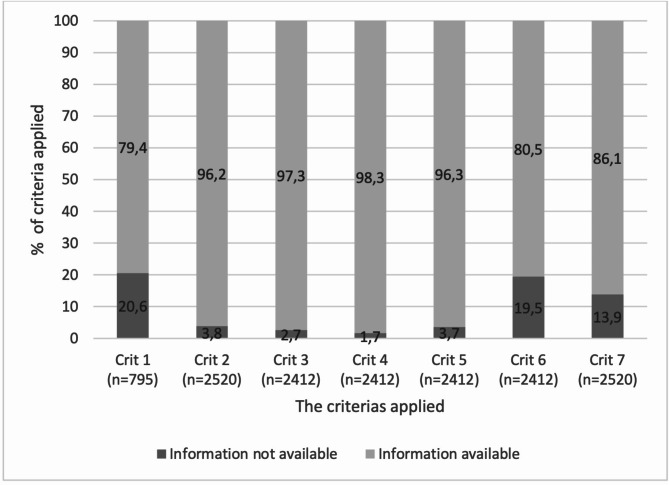
Percent of medications (*n* = 2520) in discharge summaries (*n* = 240) with requested information available Legend: Applicable criteria indicated in brackets. Crit 4. Are reasons for changes in medication use stated at discharge?, Crit5. Are generic names stated? Crit 6. Are administration forms stated? Crit 7. Are dosage strenghts stated? Crit 8. Are dosage regimes stated? Crit 9. Are indications for use stated? Crit 10. Are categories (new, changed, short course) stated? (one of the categories should be stated behind each affected medication in the medication list)

The requested medication information was available in 14,205, 91.7% of all applicable criteria, see Table 2. A total of 1682, 66.7% medications had 100% completeness of the requested information, while 33, 1.3% had none of the requested information available. Of these, 32, 1.3% medications were abscent in the medication list without any explanation, while one was in the list but without reason for change stated. Regarding the discharge summaries, 27, 11.3% contained 100% of requested information, while 3, 1.3% lacked all requested information. Highest information availability was identified for criteria 2–6 as the electronic information system adds this information automatically, see Fig. 1.

**Table 2:** Medications evaluated and total score of medication completeness discharge summaries (*n* = 240)

	0–10 percentile (*n* = 24)		10–25 percentile (*n* = 37)		25–50 percentile (*n* = 59)		50–75 percentile (*n* = 60)		75–100 percentile (*n* = 60)		Total *n* = 240	
**No of medications evaluated**												
Total number of medications, n, %	127	5.0	350	13.9	711	28.2	738	29.3	594	23.6	2520^*^	100
Mean, SD	5.3	4.6	9.5	5.9	12.1	6.2	12.3	5.3	9.9	6.3	10.5	6.1
Min/max	1	20	1	23	2	23	3	26	1	28	1.00	28
**Applicability and scores**												
Applicable criteria, n, %	829	5.4	2170	14.0	4335	28.0	4491	29.0	3658	23.6	15,483	87.8
Information available in applicable criteria, n, %	421	50.8	1870	86.2	3997	92.2	4308	95.9	3609	98.7	14,205	91.7
Mean score per medication, SD	4.1	2.08	5.8	0.48	6.0	0.44	6.1	0.26	6.2	0.34	5.9	0.94
Medications with 100% score, n, %	6	4.7	130	37.1	427	60.1	573	77.6	546	91.9	1682	66.7
Medications with 0% score, n, %	32	25.2	0	0	1	0.1	0	0	0	0	33	1.3
Proportion of requested information per discharge summary, SD	0.58	0.28	0.86	0.02	0.92	0.013	0.96	0.009	0.99	0.010	0.90	0.02
DSs with 100% completeness, n, %	0	0	0	0	0	0	0	0	27	45	27	11.3
DSs with 0% completeness, n, %	3	12.5	0	0	0	0	0	0	0	0	3	1.3

*DS* discharge summary, *SD* standard deviation

* Includes also those medications stopped during hospitalization, as this should be explained at discharge

Changes were made in 763, 31.6% medications during hospitalization, where initiation of new medication was most frequent (*n* = 532, 69.7%), followed by change either in dose, dose frequency or dose timing (*n* = 123, 16.1%) and discontinuation (*n* = 108, 14.2% ). A documentation of reason for change was given for 452, 59.% of the medications where change was indicated in the discharge summaries (Criterion 1, Fig. 1). Reason for change was most frequently documented for discontinued medications (*n* = 78,72.2%), and less frequently documented for new medications (*n* = 312,58.6%) and changed dosages (*n* = 62,50.4%).

**Fig. 2:**
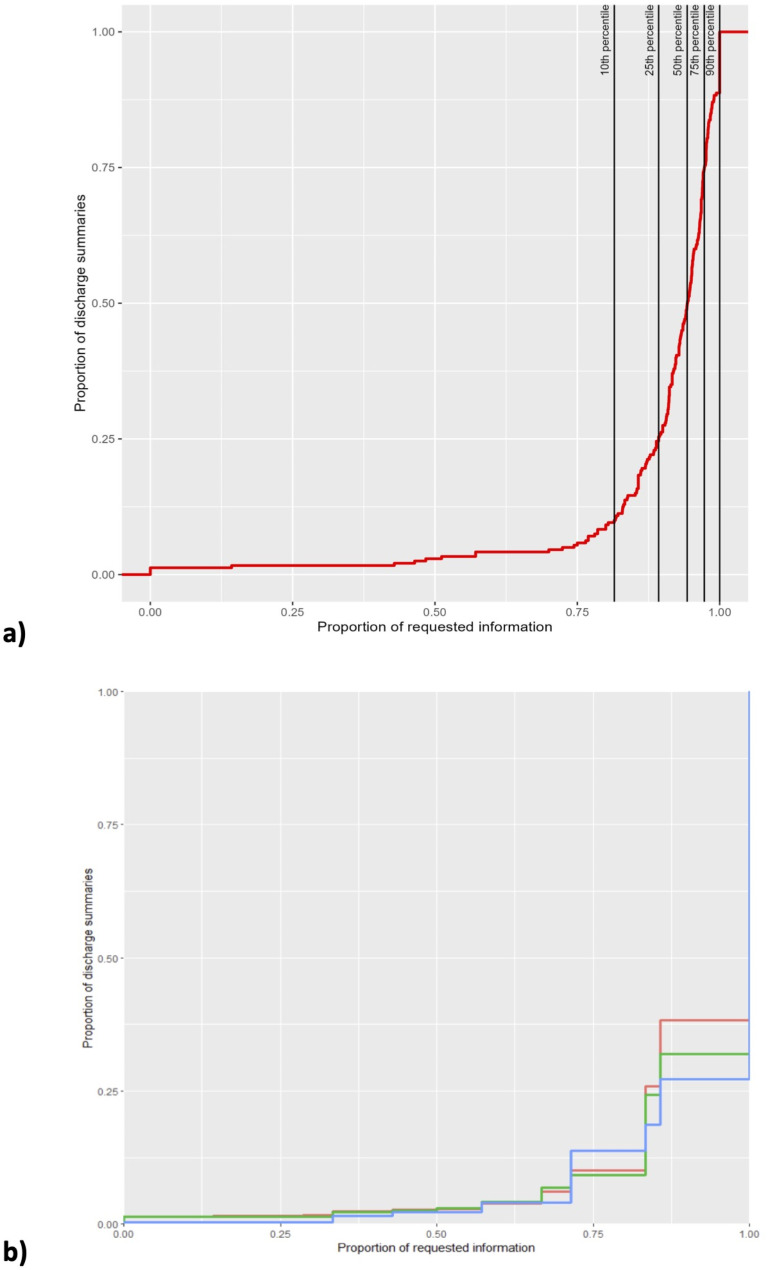
Cumulative proportion of information available on a discharge summary level (*n* = 240) Legend: Panel **a**: all medications. Panel **b**: medications used regularly (green), as needed (red) and as a short course (blue)

The proportion of information completeness on a discharge summary level varied from 0.0 to 1.0 (mean 0.904; SD 0.15, median 0.94), with 1797 (71.3%) containing ≥ 90% of the requested information, see Fig. 2a. The proportion of requested information was higher in medications used as needed compared to those used regularly. For medications used as a short course, the proportion was even lower, see Fig. 2b.

### Factors influencing completness of medication information

The quantile regression model showed that the only factor significantly influencing the proportion of medication information completeness in all quantiles was “application of electronic tool for compiling medication lists”, see Table 3 and Additional file 1. In the lowest quantile, among discharge summaries with lowest score, applying the electronic tool increases completeness with 0.89 units. The increase is not as profound in the highest quantiles, but still significant. See Additional file 2 for prediction of completeness across the qantiles by applying the electronic tool. The factors “Number of medications” is significantly associated with increased proportion of information completeness in all quantiles but Q4, but with a very low coefficient compared to applying the electronic tool. Beeing admitted from ´not home´, which in most cases is nursing homes, was associated with lower information completeness, but only in Q3.

**Table 3:** Quantile regression model showing factors associated with the proportion of medication information completeness (*n* = 240)

	Quantile 1 (Q1) – 0.1 percentile *R*^2^ = 0.576, MEA = 0.1108				Quantile 2 (Q2) – 0.25 percentile *R*^2^ = 0.355, MEA = 0.0759				Quantile 3 (Q3) – 0.5 percentile *R*^2^ = 0.208, MEA = 0.0559				Quantile 4 (Q4) – 0.75 percentile *R*^2^ = 0.098, MEA = 0.0665			
Variable	C	(95% CI)		*p*	C	(95% CI)		*p*	C	(95% CI)		*p*	C	(95% CI)		*p*
Age (years)	0.000	(−0.001	0.001)	0.426	0.000	(0.000	0.001)	0.451	0.000	(0.000	0.001)	0.223	0.000	(0.000	0.000)	0.712
Female sex^a^	0.023	(−0.011	0.057)	0.177	0.010	(−0.015	0.036)	0.420	−0.002	(−0.019	0.015)	0.833	0.002	(−0.010	0.014)	0.767
Admitted to Medical ward^b^	−0.020	(−0.060	0.021)	0.337	−0.003	(−0.033	0.027)	0.841	−0.017	(−0.037	0.003)	0.101	0.002	(−0.012	0.016)	0.800
Admitted to hospital ≤ 30 days previous (yes)	0.030	(−0.023	0.084)	0.266	0.029	(−0.011	0.069)	0.155	0.012	(−0.015	0.039)	0.372	0.004	(−0.015	0.023)	0.671
Admitted from ´not home´ ^c^	0.006	(−0.041	0.052)	0.811	−0.030	(−0.064	0.005)	0.095	−0.030	(−0.053	−0.006)	**0.013**	−0.011	(−0.027	0.006)	0.208
Number of medications	0.004	(0.001	0.008)	**0.011**	0.003	(0.001	0.006)	**0.009**	0.002	(0.000	0.003)	**0.044**	−0.001	(−0.002	0.001)	0.358
Medication reconciliation at admission (yes)^d^	0.015	(−0.049	0.079)	0.650	0.047	(−0.001	0.094)	0.057	0.044	(0.012	0.076)	**0.008**	0.002	(−0.021	0.025)	0.866
Planned admission (yes) ^e^	0.006	(−0.012	0.023)	0.523	0.010	(−0.003	0.023)	0.132	−0.002	(−0.010	0.007)	0.665	0.003	(−0.003	0.009)	0.334
Electronic tool for compilation of medication list applied (yes) ^f^	0.890	(0.817	0.962)	*P* < 0.001	0.671	(0.617	0.725)	*P* < 0.001	0.433	(0.397	0.469)	*P* < 0.001	0.231	(0.205	0.257)	*P* < 0.001
(Intercept)	−0.139	(−0.263	−0.015)	**0.028**	0.137	(0.044	0.229)	*P* < 0.001	0.500	(0.438	0.561)	*P* < 0.001	0.755	(0.711	0.799)	*P* < 0.001

*MEA* mean absolute error, *C* coefficient, *p* p-value, *Q* quantile

^a^ Reference: male sex

^b^ Reference: admitted to surgical ward

^c^ Reference: admitted from home

^d^ Reference: medication reconciliation at admission not performed

^e^ Reference: unplanned (acute) admission

^f^ Reference: no electronic tool applied, but compilated by free text

Bold = statistically significant

### Reliability testing

Reliability testing on 24 discharge summaries including 203 medications showed 86% and 96% agreement between the inter- and intra-raters, respectively. The interrater κ -value of 0.65 and intra-rater κ -value of 0.66 indicated good agreement between raters. Highest inter- and intrarater agreement was identified for criteria 1, 2 and 3, with κ -values of 0.77, 0.91, 0.91 and 0.92, 1.0. and 1.0, respectively. Lowest inter- and intrarater agreement was identified for criteria 5 and 6, with inter-rater agreement κ -values of 0.32 and 0.26 and intra-rater agreement κ-values of 0.61 and 0.36, respectively.

## Discussion

In discharge summaries from a rural hospital in mid Norway, we have identified a median proportion of information completeness of 0.94, where 71% of the discharge summaries contained ≥ 90% of the requested information. Low information completeness was mainly prevalent concerning *´explanations for change in medications during hospitalizations´* and *´indications for use of medications´*. The main factor significantly identified to increase medication completeness was the application of an electronic tool for compiling the medication list, where the coefficient for completeness increased in the range of 0.23–0.83 if the electronic tool was applied. Additionally, an increasing number of medications was also associated with higher medication completeness in most quantiles, but only with a coefficient of 0.002–0.004 per added medication.

Since 2014, the information completeness has increased dramatically [11]. Compared to other studies investigating quality of medication information in discharge summaries, the completeness identified in this study is remarkably high [6–8, 23]. One reason for this improvement is the use of the electronic medication list compiling tool, implemented in the hospital since the last audit. This tool ensures the inclusion of medication and generic names, dosages, and dosage intervals. However, the input of information such as the indication for use, explanations for prescribing changes, and coding still relies heavily on individual input from prescribers. These are areas where we identified potential for improvement.

Electronic prescribing and EMMS are increasingly being implemented world-wide. This has been shown to positively impact both processes and quality [24]. Other factors contributing to the increase in information completeness may include the increased emphasis on MedRec concurrent with the introduction of new electronic tools [12]. Additionally, employment of clinical pharmacists has increased during this period. They focus on medication appropriateness and enhancing the completeness of medication information across the hospital.

Importantly, this study does not provide insight into the quality of the medication therapy among the patients. Several studies have shown that hospital medication lists often contain discrepancies compared to what patients actually use [25, 26]. Additionally, there are drug-related problems concerning which medications patients should optimally be using and how [5, 27, 28]. Therefore, ensuring only completeness of information is insufficient. Efforts must also focus on ensuring correctness and communication of medication therapy. EMMS may be suitable for ensuring completeness, but other approaches may be needed for correctness and communication [24]. It would also be valuable to determine whether certain medications, medication groups, or patient groups are more susceptible to incomplete information. This aspect warrants further investigation in future studies.

We identified two particular areas for improvement of the medication completeness;´indication for medication use´ and ´reasons for change´, which which align with findings from other studies [6–8]. The information is most likely not automatically available in the EMMS or when using the electronic tool for compiling the medication list, as it depends on manual entry. Future EMMS should address this concern. Documenting the indication for use should be be mandatory before further processing of the prescription. Similary, when amending prescriptions, documenting reasons should be mandatory. This will ensure information availability when transferring information between care levels. Requesting prescribers to enter all relevant data can be easibly implemented into the EMMS, but it is crucial that this is adopted across all health care providers. Although research highlights the importance of accurately and timely transferring medication information across care levels for patient safety [1–3], the impact of the high completeness level observed in this study on patient safety or care continuity remains uncertain. It is also unclear which specific information is most crucial for patient safety, as its significance likely varies by medication and patient group. We recommend that future EMMS be designed to require prescribers to provide detailed information, as EMMS has been shown to reduce medication deviations at hospital discharge [29].

Interestingly, we found that an increasing number of medications was associated with higher information completeness across almost all quantiles. A plausible explanation is that prescribers pay more attention to completeness as the medication list lengthens, knowing that patients rely on the list for guidance post-discharge [30]. This observation warrants further investigation to better understand prescriber behavior and decision-making processes, especially when implementing EMMS It is crucial to recognize potential workarounds in such systems, where prescribers appear to enter required information without actually completing the task [31]. Information such as indications for use and reason for medication changes may be particularly susceptible to such workarounds, as they can be more challenging to obtain than other type of information.

### Strenghts and limitations

The main strength of this study lies in the meticulous application of criteria across a large number of medications. Since many assessments are made based on written text rather than just extractable data, automating this process is not feasible. This was further reinforced by the implementation of an application guide, which ensured procedural consistency and reduced bias resulting from the non-blinded data collection. However, we recommend that future EMMS should facilitate automatic data collection for audit and research purposes. The high inter-rater agreement indicates high validity of our results. The criteria that generated the lowest inter-rater agreement value concerned whether dosage regimes and indications for use were stated in the discharge summary. Despite a low κ-value, only 8, 0.2% and 34, 0.7% disagreements were identified for the single criteria applications, respectively. Another strength of our study is the use of nationally recognized criteria for medication information completeness, which align with international applied criteria [6, 7]. We did not strictly adhere to the scoring tool [9], as we found no documentation or rationale for weighing criteria differently, but our results are now comparable with international literature. Unfortunately, a full comparison with the 2014-study, where the scoring tool was applied per national guidelines [11], is impossible due to the introduction of new criteria. A third strength, is the low amount of missing data. Although imputation was made for the variable “living status before admission”, the imputation had only minor effect on the estimate of the coefficients while slightly increasing presicion [21]. Lastly, the regression model applied helps eliminate potential differences that might have been identified if only univariate analyses were made. This approach strengthen our findings regarding the factors that influence the medication information completeness.

Some limitations should also be considered. The cross-sectional design of this study does not establish causal relationships; future research could benefit from a longitudinal or interventional approach. Although retrospective data collection enabled us to gather data from an entire year, it potentially introduced bias due to observer interpretation, which may also have been introduced by the unblinded assessor. Nevertheless, intra-rater evaluations demonstrated high agreement. Regarding generalizability, these findings are specific to one rural Norwegian hospital, and the evaluation criteria were developed nationally. However, many hospitals, both nationally and internationally, use similar or identical electronic systems. Also, the same criteria have also been identified in international literature [6, 7]. Therefore, we believe that the findings of this study have international relevance, both for planning EMMS and for implementing electronic tools to compile discharge summaries.

## Conclusions

The overall completeness of medication information in discharge summaries from Helgelandssykehuset Mo i Rana was very high and had dramatically increased over the past decade. The use of electronic tools for compiling medication lists significantly enhances information completeness, while free-text compiled lists should be avoided. Further efforts should focus on better documenting and explaining reasons for changes in medications and indications in discharge summaries.

## Supplementary Information

Additional file 1. Quantile plots of parameters from quantile regression model.

Additional file 2. Prediction of the proportion of information available on a discharge summary level by applying the electronic tool for compiling medication lists at discharge.

## Data Availability

The datasets generated and analyzed during the current study are not publicly available because they contain information that can compromise research participants’ privacy, but some data may be available from the corresponding author on reasonable request.

## Abbreviations

EMMS: Electronic medication management system
MedRec: Medication Reconciliation
STROBE: The Strengthening the Reporting of Observational studies in Epidemiology
