# Supplementary figures and images for: Medication information completeness in discharge summaries from a Norwegian rural hospital – a cross-sectional study
Source: BMC Health Serv Res. 2025 May 1;25:634. doi: 10.1186/s12913-025-12669-x (PMC12046849; doi:10.1186/s12913-025-12669-x)

**Supplementary 1:** Quantile plots of parameters from quantile regression model


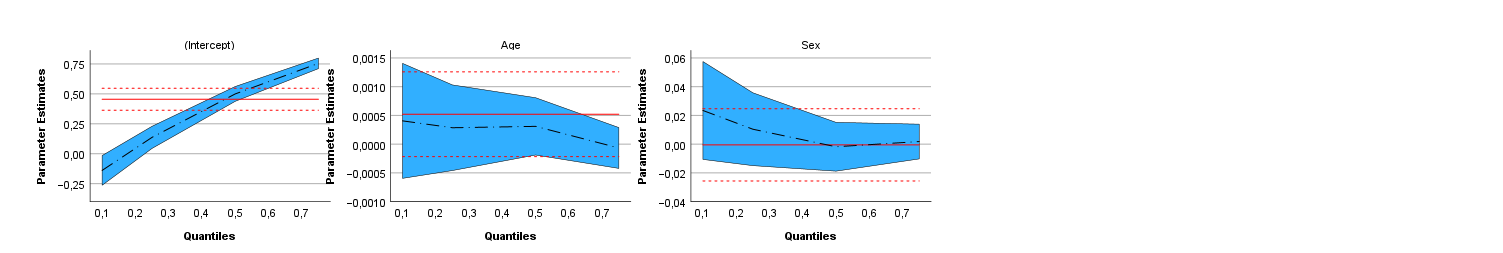

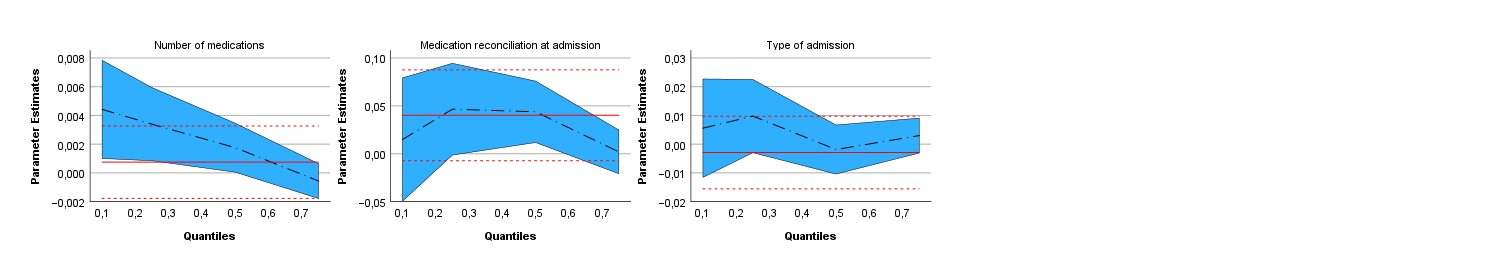

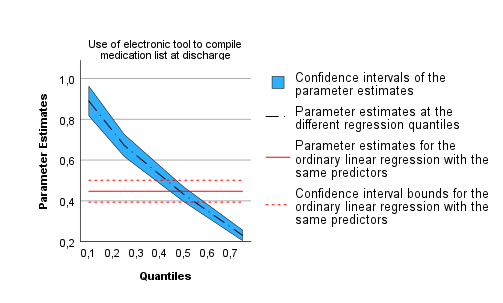

Supplement: Supplementary file 1 — Additional file 1. Quantile plots of parameters from quantile regression model. [file 12913_2025_12669_MOESM1_ESM.docx]
