# Supplementary material for: Medication information completeness in discharge summaries from a Norwegian rural hospital – a cross-sectional study
Source: BMC Health Serv Res. 2025 May 1;25:634. doi: 10.1186/s12913-025-12669-x (PMC12046849; doi:10.1186/s12913-025-12669-x)

**Supplementary 2:** Prediction of the proportion of information available on a discharge summary level by applying the electronic tool for compiling medication lists at discharge


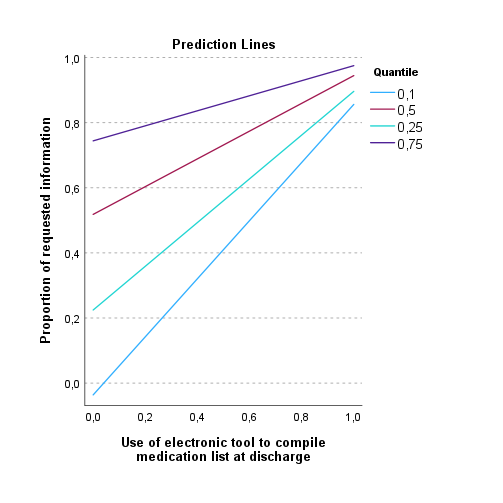

Supplement: Supplementary file 2 — Additional file 2. Prediction of the proportion of information available on a discharge summary level by applying the electronic tool for compiling medication lists at discharge. [file 12913_2025_12669_MOESM2_ESM.docx]
